# Exploring Folate Diversity in Wild and Primitive Potatoes for Modern Crop Improvement

**DOI:** 10.3390/genes6041300

**Published:** 2015-12-08

**Authors:** Bruce R. Robinson, Vidyasagar Sathuvalli, John Bamberg, Aymeric Goyer

**Affiliations:** 1 Hermiston Agricultural Research and Extension Center, Oregon State University, Hermiston, OR 97838, USA; E-Mails: Bruce.Robinson@oregonstate.edu (B.R.R.); Vidyasagar@oregonstate.edu (V.S.); 2 Department of Crop and Soil Science, Oregon State University, Corvallis, OR 97330, USA; 3 USDA/Agricultural Research Service, Sturgeon Bay, WI 54235, USA; E-Mail: john.bamberg@ars.usda.gov; 4 Department of Botany and Plant Pathology, Oregon State University, Corvallis, OR 97330, USA

**Keywords:** vitamin B_9_, folate, biofortification, potato, *Solanum tuberosum*, *andigenum*, *vernei*

## Abstract

Malnutrition is one of the world’s largest health concerns. Folate (also known as vitamin B_9_) is essential in the human diet, and without adequate folate intake, several serious health concerns, such as congenital birth defects and an increased risk of stroke and heart disease, can occur. Most people’s folate intake remains sub-optimal, even in countries that have a folic acid food fortification program in place. Staple crops, such as potatoes, represent an appropriate organism for biofortification through traditional breeding based on their worldwide consumption and the fact that modern cultivars only contain about 6% of the daily recommended intake of folate. To start breeding potatoes with enhanced folate content, high folate potato material must be identified. In this study, 250 individual plants from 77 accessions and 10 *Solanum* species were screened for their folate content using a tri-enzyme extraction and microbial assay. There was a 10-fold range of folate concentrations among individuals. Certain individuals within the species *Solanum tuberosum* subsp*. andigenum*, *Solanum vernei* and *Solanum boliviense* have the potential to produce more than double the folate concentrations of commercial cultivars, such as Russet Burbank. Our results show that tapping into the genetic diversity of potato is a promising approach to increase the folate content of this important crop.

## 1. Introduction

Tetrahydrofolate (THF or vitamin B9) and its derivatives, commonly known as folates, are essential micronutrients in the human diet. Essential micronutrients refer to nutrients that are required by humans in small amounts from the diet, and if lacking, symptoms of deficiency occur. Folates are crucial for many cellular functions, including nucleic acid synthesis, the metabolism of the amino acids methionine, glycine, serine, histidine and glutamic acid and methylation reactions [1,2,3,4]. Because humans cannot synthesize folate *de novo* and must obtain it from their diet, adequate folate intake is critical for overall nutritional health. Chronic folate deficiency has been linked to several serious diseases, such as congenital birth defects, anemia, increased risk of stroke, certain types of cardiovascular diseases and cancers [5,6,7]. Low folate levels have also been linked to impaired cognitive performance and depression [8,9,10]. Research suggests that folic acid supplementation or adequate folate intake can improve the effectiveness of anti-depression medications [9,10]. Unfortunately, folate intake remains suboptimal, even in countries that have implemented industrial folic acid fortification of staple foods [11,12,13]. A complementary strategy to folic acid food fortification is biofortification. Biofortification is the process by which the nutritional quality of food crops is improved through biological means, such as conventional breeding (World Health Organization). It differs from food fortification, which is a manual post-harvest processing method, in that it enhances the plant’s natural ability to produce increased amounts of nutrients. There are two primary approaches to biofortification, genetic engineering and traditional breeding, both of which could complement folic acid food fortification [14]. Traditional breeding has the advantage of being more accepted by the general public compared to genetically-engineered crops. Traditional breeding also offers an alternative strategy to genetic engineering strategies, which although successful for some crops, have failed in potato up to now [14]. In either case, biofortification of crops may be able to decrease folate deficiencies in at-risk populations and in less developed regions of the world, where the funding and infrastructure for industrial folic acid food fortification does not exist.

Potatoes represent an appropriate vehicle for enhanced nutrition for several reasons. Their cultivation distribution is enormous, accounting for over 300 million tonnes grown in over 150 countries in 2014 [15]. China and India are now the world’s largest producers of potatoes, and their production is still increasing [16]. Half of the world’s root and tuber crop are potatoes, and they are considered to be the fourth most important food crop in the world, third in terms of human consumption [15,17]. It is estimated that over one billion people worldwide consume potatoes regularly [15]. Consumption is rapidly growing in Africa and Latin America. Potatoes have the ability to produce more calories and tonnage per acre than any other crop [16]. Therefore, potatoes may be the best option to help feed a growing population in a world where food security is a serious concern.

Independent studies in several countries have reported on the contribution of potatoes to dietary folate intake. A study of a randomly-selected Finnish population found that potatoes account for 10% of the total folate intake in middle-aged men and represent one of the best sources for folate in the diet [18,19]. A similar study in The Netherlands found that potatoes account for 7% of the daily folate intake for adults and were the third greatest contributor to dietary folate intake [12]. A Greek nutritional study found that people who had an increased intake of potatoes were at a significantly decreased risk of low folate serum levels [20].

Previously, we have shown that modern potato genotypes have a relatively narrow range of folate concentrations, ranging between 400 and 1300 ng·g^−1^ folate dry weight, while significantly higher folate concentrations could be found in some wild species and primitive cultivars, with concentrations two- to four-fold higher than in commercial varieties, such as Russet Burbank [21,22]. Species, such as *S. boliviense*, *S. vernei* and *S. tuberosum* subsp. *andigenum*, have accessions that could be promising sources of high folate trait [22]. In this study, the screening effort was expanded within these promising species, as well as into other wild and primitive cultivated potato species that had not been screened in order to further evaluate the natural variation of folate content in potatoes and to identify germplasm appropriate for breeding high folate trait(s) into modern cultivars.

## 2. Experimental Section

### 2.1. Chemicals and Reagents

Folate (5-formyltetrahydrofolate (5-formyl-THF)) standard was obtained from Schircks Laboratories (Jona, Switzerland). Rat plasma conjugase was obtained from Rockland Laboratories (Limerick, PA, USA) and was dialyzed before use, as described previously [23]. Difco folic acid casei medium and *Lactobacilli* Broth AOAC were from Becton, Dickinson, and Company (Sparks, MD, USA). All other chemicals (protease, α-amylase) were obtained from Sigma Chemical.

### 2.2. Potato Material

Folate screening for wild and primitive cultivated species included 250 individual plants from 77 accessions and 10 species (*S. stipuloideum*, *S. chacoense* subsp. *chacoense*, *S. candolleanum*, *S. acaule*, *S. demissum*, *S. microdontum*, *S. okadae*, *S. tuberosum* subsp. *andigenum*, *S. boliviense*, *S. vernei*) (Table 1). The species *S. tuberosum* subsp. *andigenum*, *S. boliviense* and *S. vernei* were selected based on previous data, which showed that they could contain accessions with high folate [22]. The species *S. stipuloideum*, *S. candolleanum*, *S. acaule*, *S. demissum*, *S. microdontum* and *S. okadae* were selected because no or very few accessions within these species had been previously evaluated [21,22]*. S. chacoense* subsp. *chacoense* was evaluated because it is one of the most widely distributed wild potato species. Russet Burbank, a commercial variety largely grown in North America, was used as the standard [22]. Seeds of wild and primitive cultivated species were obtained from the U.S. potato gene bank (USDA Agricultural Research Service Germplasm Resource Information Network (GRIN), www.ars-grin.gov). Seeds were soaked in GA3 at 1000 mg/L overnight before planting to Metro-mix in June 2014. When plantlets reached about 8 cm high, they were transplanted in 8 cm square individual pots containing Sunshine^®^ LA4 P. All-purpose fertilizer 20-20-20 was applied at 200 mg/L once a week until senescence. Plants were watered twice a week until senescence. Vines were killed on 31 October 2014, and tubers were harvested on 11 November. Greenhouse temperature was set at 21 °C day time and 15 °C night time. Supplemental light was provided for 14 h per day from a mixture of 400-Watt high pressure sodium and 1000-Watt metal halide lamps.

**Table 1:** Classification, available accessions, ploidy level, accessions evaluated and their geographic origin. GRIN, Germplasm Resource Information Network.

Species: Spooner Classification [24]	Species: Old Classification	Number of Accessions Available from GRIN	Ploidy Level	Number of Accessions Tested	PI Number Tested	Origin
*S. stipuloideum*	*S. circaeifolium*	14	2×	3	498116	Cochabamba, Bolivia
					498120	Santa Cruz, Bolivia
					545974	La Paz, Bolivia
*S. chacoense*	*S. chacoense* subsp. *chacoense*	174	2×	2	197760	?
					320293	Salta, Argentina
*S. candolleanum*	*S. bukasovii*	176	2×	3	265863	Puno, Peru
					365321	Huanuco, Peru
					458379	Apurimac, Peru
*S. acaule*	*S. acaule f. acaule*	424	4×	3	175395, 472661	Argentina
					473481	Huancavelica, Peru
*S. demissum*	*S. demissum*	164	6×	3	160208	Mexico
					230589	Huanuco, Peru
					498232	Apurimac, Peru
*S. microdontum*	*S. microdontum* subsp. *microdontum*	116	2×	2	458355	Jujuy, Argentina
					498123	Chuquisaca, Bolivia
*S. okadae*	*S. venturii*	16	2×	1	458368	Salta, Argentina
	*S. okadae*		2×	2	498130	Cochabamba, Bolivia
					320327	Salta, Argentina
*S. tuberosum* subsp. *andigenum*	*S. stenotomum* subsp. *stenotomum*	1006	2×	2	195204	Cuzco, Peru
					283141	Colombia
	*S. phureja* subsp. *phureja*		2×	3	320355, 320377	Narino, Colombia
					225710	Cauca, Colombia
	*S. tuberosum* subsp. *andigenum*		4×	3	546023	Potosi, Bolivia
					607886	Cuzco, Peru
					281034	Mexico
*S. boliviense*	*S. megistacrolobum*	222	2×	26	283082	Bolivia
					283133	Ecuador
					275149, 435077, 500029, 500030	Salta, Argentina
					458347, 458348, 473110, 473112, 473113, 473124, 473129, 473130, 473138, 473141, 473144, 473149, 473160, 558094	Jujuy, Argentina
					545899, 568986	Tarija, Bolivia
					597689	Oruro, Bolivia
					597705, 597706, 597736	Potosi, Bolivia
*S. vernei*	*S. vernei* subsp*. vernei*	36	2×	18	320332	Catamarca, Argentina
					230468, 458373, 473308	Tucuman, Argentina
					458374, 473306, 473310, 473311, 500045, 500062, 500063, 500065, 558147, 558148	Salta, Argentina
					500067, 500069, 558149, 558150	Jujuy, Argentina
	*S. vernei* subsp*. ballsii*		2×	5	458369, 473303	Jujuy, Argentina
					458370, 458371, 458372	Salta, Argentina
	*S. vernei*		2×	1	500066	Jujuy, Argentina

One to 4 individual plants per accession, with a minimum of 3 in most instances, were grown. One individual plant is a plant from one botanical seed. A representative set of tubers from one individual plant was pooled and processed together as follows. Tubers were left with skin intact, washed with cold water in a strainer, weighed and then flash-frozen with liquid nitrogen before storage at −80 °C. A few tubers from each genotype were stored at 4 °C as back-up for re-planting. Frozen samples were then lyophilized in a freeze-dryer (VirTis Benchtop K) (vacuum pressure <100 mTorr) for two to three days. Dried samples were weighed, and the initial moisture content was calculated by the weight difference before and after freeze-drying potato samples [22]. Removal of water from tuber samples allows for a more consistent comparison of vitamin content among samples, because moisture content varies greatly in these materials (68% to 82%). Samples (*i.e.*, one sample is made of several tubers from one individual plant) were then ground to a fine powder with a Waring blender and transferred to scintillation vials for long-term storage at −80 °C.

### 2.3. Folate Analysis

Folates were extracted by using a tri-enzyme extraction method, as previously published [21,22]. Potato samples (100 mg) were homogenized in 15-mL Falcon tubes containing 10 mL of extraction buffer consisting of 50 mM HEPES/50 mM CHES, pH 7.85, 2% (*w/v*) sodium ascorbate and 10 mM β-mercaptoethanol and deoxygenated by flushing with nitrogen. Once homogenized, samples were boiled for 10 min and cooled immediately on ice in a covered cooler. The homogenate was then treated with protease (≥14 units) and incubated for 2 h at 37 °C, boiled again for 5 min and cooled immediately in a covered cooler of ice. The samples were then treated with α-amylase (≥800 units) and rat plasma conjugase in large excess (0.5 mL/sample), incubated for 3 h at 37 °C, boiled again for 5 min and cooled immediately in a covered cooler of ice. After centrifugation at 3000 g for 10 min, the supernatant was transferred to a new tube. The residue was re-suspended and homogenized in 5 mL of extraction buffer, re-centrifuged for 10 min, and the supernatant was recovered. Supernatants were then combined and the samples’ volume adjusted to 20 mL with extraction buffer. Aliquots of each sample were transferred to 1.5-mL microcentrifuge tubes, flushed with nitrogen and stored at −80°C until analysis by the microbiological assay. Controls containing all reagents, but potato samples, were used to determine the amount of any residual folates in the reagents. There were no detectable folates in any of the reagents used.

Folate concentrations were measured by microbiological assay using *Lactobacillus rhamnosus*. *L. rhamnosus* (ATCC 7469) cultures were obtained from the American Type Culture Collection (Manassas, VA, USA). Glycerol cryoprotected cells of *L. rhamnosus* were prepared as described previously [25]. Assays were performed in 96-well plates (Falcon microtiter plates). Wells contained growth medium supplemented with folate standards or potato extracts, each plated in triplicate. Bacterial growth was measured at 630 nm after 18 h, 21 h and 24 h of incubation at 37 °C. The 24-h reading was usually used for analysis unless saturation was reached, in which case, the 21-h reading was used. All measurements were made with a BioTek Instrument EL 311 SX microplate auto-reader (BioTekInstrument, Winooski, VT, USA), analyzed with the KCJr EIA application software (BioTekInstrument, Winooski, VT, USA) and compiled in Microsoft Excel. Final results were calculated by reference to a standard curve using 5-formyl-THF and expressed as nanograms of folate per gram of dry sample.

A large batch of dried potato powder was prepared from tubers of *Solanum pinnatisectum* PI 275233 and was used as the reference material. Each batch of extractions contained 18 samples plus the reference material. Values obtained for samples were normalized to values obtained for the reference material. The average folate concentration of the reference material across all of the extractions was 1105 ± 76 ng·g^−1^ DW. All calculations were performed with standard function settings in Microsoft Excel.

### 2.4. Statistical Analysis

One-way analysis of variance (ANOVA) was performed to compare normalized mean values of folate content in all species. The only species that was significantly different from all other species at a *p*-value ≤ 0.001 was *S. vernei*. All statistical analysis was performed with R in R-Studio with the “stats” package linear regression and ANOVA functions.

## 3. Results

**Figure 1:**
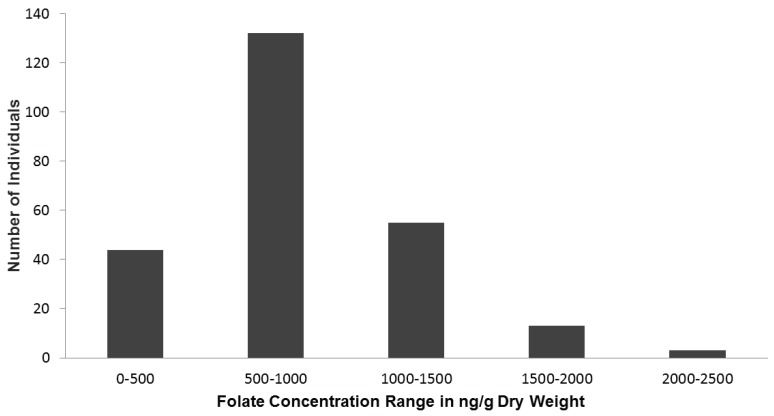
Histogram of number of individuals within folate concentration brackets.

Overall, folate concentrations ranged from 221 ± 19 to 2336 ± 285 ng·g^−1^ dry weight (Table 2), representing a 10.5-fold difference between the lowest and highest folate concentration. The majority of individuals (55% of all individuals tested) had folate concentrations between 500 and 1000 ng·g^−1^ dry weight, including the modern variety Russet Burbank (Figure 1).

**Table 2:** Folate concentration (mean ± SE) in ng·g^−1^ dry matter per accession. In bold are individuals with folate concentrations higher than 1500 ng·g^−1^ dry weight.

Plant Introduction Number	Species	Number of Individuals Tested	Individual Measurements	Mean ± SE	% DM
R. Burbank	*S. tuberosum* subsp. *tuberosum*	3	1276-915-929	1040 ± 118	26
498116	*S. stipuloideum*	3	907-1115-1118	1046 ± 57	25
498120	*S. stipuloideum*	3	304-553-532	463 ± 65	25
545974	*S. stipuloideum*	4	415-824-1119-882	810 ± 127	23
197760	*S. chacoense*	3	591-653-653	632 ± 17	36
320293	*S. chacoense*	4	478-240-1198-408	581 ± 183	37
265863	*S. candolleanum*	3	1367-508-481	786 ± 206	25
365321	*S. candolleanum*	1	918	918 ± n.d.	19
458379	*S. candolleanum*	1	1023	1023 ± n.d.	19
175395	*S. acaule*	4	461-562-562-1017	651 ± 108	22
472661	*S. acaule*	4	480-490-517-1014	625 ± 113	23
473481	*S. acaule*	2	632-757	695 ± 44	25
160208	*S. demissum*	4	749-455-487-556	562 ± 57	23
230589	*S. demissum*	2	410-631	520 ± 78	22
498232	*S. demissum*	4	760-737-669-455	655 ± 60	30
458355	*S. microdontum*	3	703-694-650	682 ± 13	32
498123	*S. microdontum*	2	913-767	840 ± 51	36
320327	*S. okadae*	3	876-548-629	684 ± 80	35
458368	*S. okadae*	3	611-1317-991	973 ± 167	34
498130	*S. okadae*	3	723-660-806	730 ± 34	38
195204	*S. tuberosum* subsp*. andigenum*	4	410-836-499-794	635 ± 92	24
225710	*S. tuberosum* subsp*. andigenum*	1	**2337**	2337 ± n.d.	22
281034	*S. tuberosum* subsp*. andigenum*	4	565-1030-1126-506	807 ± 137	18
283141	*S. tuberosum* subsp*. andigenum*	3	468-457-711	545 ± 68	18
320355	*S. tuberosum* subsp*. andigenum*	2	853-1400	1126 ± 193	28
320377	*S. tuberosum* subsp*. andigenum*	2	**2198**-1038	1618 ± 410	17
546023	*S. tuberosum* subsp*. andigenum*	4	985-700-333-626	661 ± 116	21
607886	*S. tuberosum* subsp*. andigenum*	4	404-553-622-361	485 ± 53	21
275149	*S. boliviense*	4	566-561-602-515	561 ± 15	24
283082	*S. boliviense*	1	934	934 ± n.d.	23
283133	*S. boliviense*	4	891-1102-1097-351	860 ± 153	27
435077	*S. boliviense*	3	421-652-630	568 ± 60	26
458347	*S. boliviense*	3	779-1393-525	899 ± 210	21
458348	*S. boliviense*	4	585-759-666-679	672 ± 31	23
473110	*S. boliviense*	4	362-456-651-611	520 ± 58	18
473112	*S. boliviense*	4	517-897-450-688	638 ± 87	20
473113	*S. boliviense*	1	869	869 ± n.d.	20
473124	*S. boliviense*	4	630-547-456-610	561 ± 34	20
473129	*S. boliviense*	4	816-997-722-584	780 ± 75	21
473130	*S. boliviense*	4	411-526-512-751	550 ± 62	24
473138	*S. boliviense*	4	1265-787-512-745	827 ± 137	21
473141	*S. boliviense*	4	460-524-630-385	500 ± 45	23
473144	*S. boliviense*	4	473-355-222-449	375 ± 49	22
473149	*S. boliviense*	4	628-523-826-780	689 ± 60	24
473160	*S. boliviense*	4	557-352-706-347	491 ± 75	22
500029	*S. boliviense*	4	684-461-610-622	594 ± 41	26
500030	*S. boliviense*	4	541-494-575-671	634 ± 32	24
545899	*S. boliviense*	4	647-426-1033-780	721 ± 110	22
558094	*S. boliviense*	3	350-473-542	455 ± 46	21
568986	*S. boliviense*	2	684-888	786 ± 72	19
597689	*S. boliviense*	4	570-1099-723-822	804 ± 96	22
597705	*S. boliviense*	4	332-749-366-707	539 ± 95	20
597706	*S. boliviense*	4	484-567-551-623	556 ± 25	23
597736	*S. boliviense*	4	713-**1947**-539-777	994 ± 279	33
230468	*S. vernei*	4	1377-1072-1416-**1911**	1444 ± 150	24
320332	*S. vernei*	4	1137-846-**1985**-1105	1268 ± 215	28
458369	*S. vernei*	2	1197-1002	1099 ± 69	22
458370	*S. vernei*	4	1207-1062-1073-817	1040 ± 70	20
458371	*S. vernei*	4	**1940**-786-881-**1601**	1302 ± 242	22
458372	*S. vernei*	4	1450-**1801**-1023-1110	1346 ± 154	23
458373	*S. vernei*	2	1316-1145	1230 ± 60	26
458374	*S. vernei*	4	891-838-851-908	872 ± 14	25
473303	*S. vernei*	3	**1623**-1099-774	1165 ± 202	21
473306	*S. vernei*	3	**1968**-1307-**1703**	1659 ± 157	18
473308	*S. vernei*	1	1117	1117 ± n.d.	25
473310	*S. vernei*	3	649-973-1058	893 ± 102	21
473311	*S. vernei*	3	**1589**-1309-1122	1340 ± 111	23
500045	*S. vernei*	2	1361-826	1093 ± 189	26
500062	*S. vernei*	2	1287-818	1053 ± 166	25
500063	*S. vernei*	4	469-1282-776-725	813 ± 147	22
500065	*S. vernei*	3	829-1105-835	923 ± 74	25
500066	*S. vernei*	3	1178-**1722**-1372	1424 ± 112	20
500067	*S. vernei*	4	1219-1370-961-1035	1146 ± 280	22
500069	*S. vernei*	3	970-948-1294	1070 ± 91	24
558147	*S. vernei*	2	1117-1150	1133 ± 12	23
558148	*S. vernei*	4	853-974-1312-959	1024 ± 86	26
558149	*S. vernei*	4	1268-**2211**-**1688**-1355	1630 ± 185	24
558150	*S. vernei*	2	909-**1620**	1264 ± 252	22

About 40% of individuals had folate concentrations below 500 or between 1000 and 1500 ng·g^−1^ dry weight. The remaining 10% had folate concentrations above 1500 ng·g^−1^ dry weight, with thirteen individuals between 1500 and 2000 ng·g^−1^ dry weight and three above 2000 ng·g^−1^ dry weight (Figure 1). In most cases, a minimum of three individual plants per accession were evaluated for folate (Table 2).

**Figure 2:**
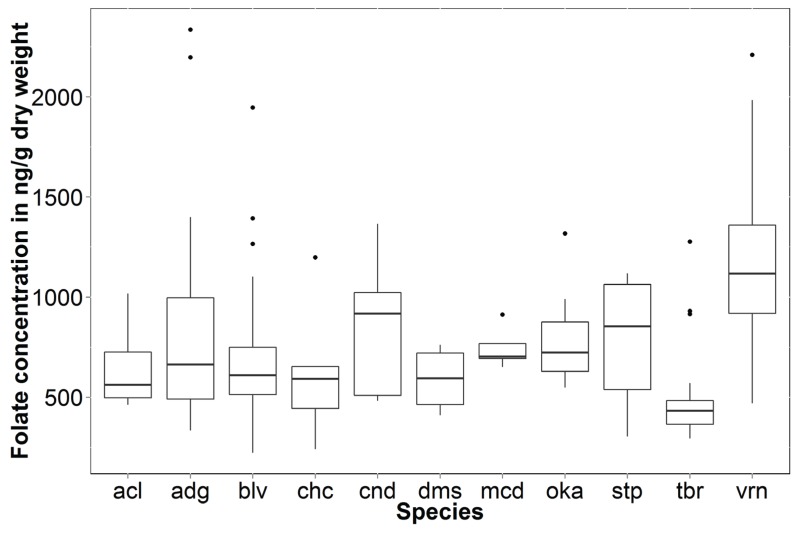
Box and whisker plot of folate concentrations by species. acl, *S. acaule*; adg, *S. tuberosum* subsp. *andigenum*; blv, *S. boliviense*; chc, *S. chacoense* subsp. *chacoense*; cnd, *S. candolleanum*; dms, *S. demissum*; mcd, *S. microdontum*; oka, *S. okadae*; stp, *S. stipuloideum*; tbr, *S. tuberosum*; vrn, *S. vernei*. For *S. tuberosum*, data for two varieties, Russet Burbank and Yukon Gold, each in three biological replicates, were used.

Each accession showed different levels of variability between individuals, with some accessions displaying a low level of variability (e.g., PI 197760), while other accessions had individuals with up to a five-fold folate concentration range (e.g., PI 320293). Two individuals with folate concentrations above 2000 ng·g^−1^ dry weight were from the accessions PI 225710 and PI 320377, both from the species *S. tuberosum* subsp. *andigenum*. Overall, about 25% of all individuals from the species *S. tuberosum* subsp. *andigenum* had folate concentrations above 1000 ng·g^−1^ dry weight (Table 2 and Figure 2). For *S. boliviense*, one individual within the accession PI 597736 contained folate concentrations of 1947 ng·g^−1^ dry weight. Only 7.5% of all individuals from the species *S. boliviense* had folate concentrations above 1000 ng·g^−1^ dry weight (Table 2 and Figure 2). For *S. vernei*, one individual from the accession PI 558149 had folate concentrations above 2000 ng·g^−1^ dry weight, and seven individuals from six different accessions (PI 320332, PI 458371, PI 458372, PI 473306, PI 500066 and PI 558149) contained folate concentrations greater than 1700 ng·g^−1^ dry weight. Over 66% of all individuals from this species had folate concentrations above 1000 ng·g^−1^ dry weight (Table 2 and Figure 2). Amongst the species *S. stipuloideum*, *S. candolleanum*, *S. acaule*, *S. demissum*, *S. microdontum*, *S. okadae* and *S. chacoense* subsp. *chacoense*, no individual had folate concentrations above 1500 ng·g^−1^ dry weight (Table 2 and Figure 2). *S. demissum* had the lowest maximum folate concentration (760 ng·g^−1^ dry weight), while *S. candolleanum* had the highest (1367 ng·g^−1^ dry weight) (Figure 2).

**Figure A1:**
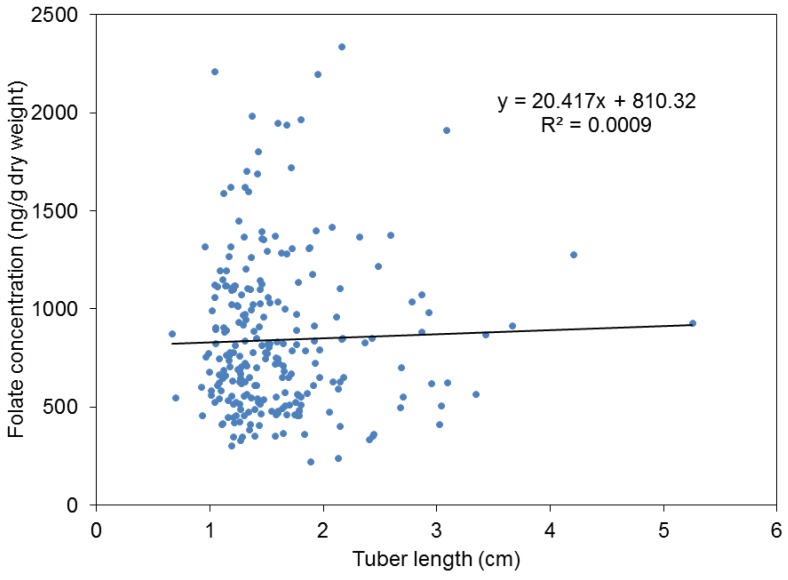
Relationship between folate concentration and tuber length.

As previously demonstrated [21,26], the peel contains a substantially higher amount of folates than the potato flesh. Because tubers from wild and primitive species are usually small, the relatively higher contribution of the peel could be responsible for at least part of the high folate concentrations observed. However, when the tuber length of five to six representative tubers for each individual tested in this study was plotted against folate concentrations, the coefficient of correlation *r* was 0.03 (Figure A1). These results indicate that tubers of a similar size can have very different folate content and show that a tuber with a relatively higher amount of peel does not necessarily contain a higher amount of folates.

## 4. Discussion

This study shows that there is an enormous amount of genetic diversity within the germplasm that was evaluated for folate content. It also shows that *S. tuberosum* subsp. *andigenum*, *S. boliviense* and *S. vernei* all contain individuals that have the ability to produce and accumulate significantly higher concentrations of folate (over two-fold) in their tubers than a modern commercial variety, such as Russet Burbank. These individuals are promising materials for breeding potato with high folate content. *S. tuberosum* subsp. *andigenum*, *S. vernei* and *S. boliviense* were selected for evaluation based on a previous study with fewer individuals that showed that high folate concentrations could be found within these species [22]. Of particular interest is the accession PI 225710 from the species *S. tuberosum* subsp. *andigenum* from which we found the highest folate concentrations (>2000 ng·g^−1^ dry weight). Another individual (clone named RN018.03) from the same accession that contained folate concentrations above 2000 ng·g^−1^ dry weight had previously been identified [22]. Therefore, this accession may be a good source of high folate individuals. However, more individuals will need to be evaluated to confirm this hypothesis. One individual from the accession PI 320377 from the species *S. tuberosum* subsp. *andigenum* also had folate concentrations above 2000 ng·g^−1^ dry weight. Other individuals from this accession were previously reported in the low to mid-range folate levels [22]. The highest folate concentration found within the species *S. boliviense* was in an individual from the accession PI 597736 (1947 ng·g^−1^ dry weight). This accession had previously provided individuals with average folate concentrations above 3000 ng·g^−1^ dry weight [22] and may also be a good source of high folate individuals. It should be noted that the concentration of 3000 ng·g^−1^ dry weight was found in tubers that were stored at a cold temperature for six months. We have found that cold storage could significantly increase folate concentrations [21]. Re-evaluation of one of these individuals has shown more modest folate concentrations (1500 to 2000 ng·g^−1^ dry weight) in freshly-harvested tubers. Five individuals from the species *S. vernei* had folate concentrations above 1900 ng·g^−1^ dry weight, including one individual from the accession PI 230468. We had previously found individuals from this accession with average folate concentrations above 1500 ng·g^−1^ dry weight [22]. The accession PI 558149 had one individual with folate concentrations above 2200 ng·g^−1^ dry weight and an average folate concentration for four individuals higher than 1600 ng·g^−1^ dry weight. Therefore, these accessions may be other good sources of high folate individuals. The species *S. vernei* also had a large number of individuals with folate concentrations above 1000 ng·g^−1^ dry weight (49 out of 74 individuals). By comparison, *S. boliviense* only had seven out of 93 individuals with folate concentrations above 1000 ng·g^−1^ dry weight. *S. vernei* may therefore be a good species to further evaluate. No high folate (>1500 ng·g^−1^ dry weight) individuals were identified in the other species evaluated in this study (*S. stipuloideum*, *S. candolleanum*, *S. acaule*, *S. demissum*, *S. microdontum*, *S. okadae* and *S. chacoense* subsp. *chacoense*). Although one cannot preclude that high folate individuals could still be identified by extending the screening, our results also indicate that these species may not be the best genetic pool to screen for high folate individuals.

Although our data indicate that some accessions and some species may be better sources of high folate individuals than others, our results also illustrate the high degree of variability within accessions and species. Thus, until a larger number of individuals are being evaluated within each accession and species, it is currently difficult to pinpoint, with high confidence, a specific accession and/or species for high folate content. It should be emphasized that screening wild or primitive potato species for folate is very tedious. First, folate analyses are very time consuming. Second, tubers from wild and primitive species are difficult to produce; they are very small, most often between the size of a marble and a golf ball, and most species do not tuberize in the field, so they have to be grown in winter greenhouses or crossed with adapted cultivated forms. It would therefore be very helpful to identify predictors of high folate tubers. To this end, we are currently genotyping for single nucleotide polymorphisms (SNPs) a segregating population from a cross between a high folate *S. boliviense* individual (accession PI 597736) with a diploid *S. tuberosum* clone. We are also examining the possibility of correlation between leaf, seed and tuber folate content and other tuber characteristics, such as pH, which all could decrease the time and effort needed to screen a large number of individuals.

Once identified, high folate individuals should be used to introgress the high folate trait(s) into *S. tuberosum* tetraploid cultivars adapted for commercial production. The species evaluated in this study have different ploidy levels (2×, 4× or 6×) (Table 1) and belong to different crossability groups. *S. tuberosum* subsp. *andigenum* is cultivated like *S. tuberosum* cultivars and very easy to introgress. We have obtained hybrids from a cross between a high folate individual from the accession PI 225710 and a diploid *S. tuberosum* clone. These hybrids were grown in the field and are currently being evaluated for folate. Both *S. boliviense* and *S. vernei*, which had high folate individuals, should be very easy to move into the cultivar genepool by 2n gametes or by making 4× versions of the wild species.

If the high folate trait(s) are successfully introgressed into modern potatoes, such as Russet Burbank, new commercial potato cultivars could contain double the amount of folate compared to currently-grown cultivars. Based on the current per capita consumption of 50 kg per year, or 137 g per day in the United States, such a potato would provide around 11% of the recommended daily need of 400 μg, assuming 20% dry matter and 80% retention during cooking. The highest folate concentrations measured in this study (e.g., >2000 ng/g dry weight or >400 ng/g fresh weight, assuming 20% dry matter) were higher than those found in lettuce, snap beans and oranges (~300 to 380 ng/g fresh weight, according to the USDA Nutrient Database), for instance, but still much lower than high folate sources, such as beans, lentils and spinach (~2000 to 6000 ng/g fresh weight). The increase obtained by genetic means could be further increased by optimizing the time of harvest, as young tubers contain up to two-fold the amount found in mature tubers [27], the storage conditions, as folates accumulate up to two-fold in tubers stored at cold temperature [21], and the cooking method, as studies show the retention rate fluctuating between 50% and 110% depending on the cooking method and the cultivar [28]. The reported folate retention rate for processed potatoes (*i.e.*, French fries) is usually high (>75%) compared to boiled potatoes, for instance. This may be due to the shorter cooking time and the insolubility of folates in cooking oil during processing. In addition, potatoes destined for processing are often stored for several months at a cold temperature. With the increasing consumption of processed potatoes in the United States, introgression into a processing cultivar may have the most impact on the U.S. population’s folate intake.

In the future, screening of additional individuals within promising accessions and species should help determine whether screening should focus on these specific accessions and species. Future research should also investigate the stability of the high folate genotypes across environments and the heritability of the high folate trait(s). Finally, the development of fast and easy-to-use predictors for high folate, such as molecular markers, is essential to accelerate the screening of potato genotypes and to assess the full potential of the potato genetic diversity.

## 5. Conclusions

In conclusion, this study highlights the genetic potential of potato for folate biofortification and illustrates the importance of collecting and evaluating exotic germplasm for improving potato varieties.
